# Construction of a Prognostic Model for Mitochondria and Macrophage Polarization Correlation in Glioma Based on Single‐Cell and Transcriptome Sequencing

**DOI:** 10.1111/cns.70083

**Published:** 2024-11-03

**Authors:** Pengyu Chen, Heping Wang, Yufei Zhang, Siyao Qu, Yulian Zhang, Yanbo Yang, Chuanpeng Zhang, Kun He, Hanhan Dang, Yang Yang, Shaoyi Li, Yanbing Yu

**Affiliations:** ^1^ China–Japan Friendship Hospital (Institute of Clinical Medical Sciences) Chinese Academy of Medical Sciences and Peking Union Medical College Beijing China; ^2^ Department of Neurosurgery China–Japan Friendship Hospital Beijing China; ^3^ Department of Biochemistry & Molecular Biology, State Key Laboratory of Common Mechanism Research for Major Diseases Institute of Basic Medical Sciences Chinese Academy of Medical Sciences & School of Basic Medicine Peking Union Medical College Beijing China; ^4^ Department of Medical Genetics China Medical University Shenyang Liaoning China; ^5^ Department of Neurosurgery Peking University China–Japan Friendship School of Clinical Medicine Beijing China; ^6^ Department of Neurosurgery, the First Affiliated Hospital of USTC, Division of Life Sciences and Medicine University of Science and Technology of China Hefei Anhui China; ^7^ Department of Neurosurgery Shengjing Hospital of China Medical University Shenyang Liaoning China

**Keywords:** glioma, immune microenvironment, macrophage polarization, mitochondria, risk model, single‐cell RNA‐seq analysis

## Abstract

**Background:**

Numerous diseases are associated with the interplay of mitochondrial and macrophage polarization. However, the correlation of mitochondria‐related genes (MRGs) and macrophage polarization‐related genes (MPRGs) with the prognosis of glioma remains unclear. This study aimed to examine this relationship based on bioinformatic analysis.

**Methods:**

Glioma‐related datasets (TCGA‐GBMLGG, mRNA‐seq‐325, mRNA‐seq‐693, GSE16011, GSE4290, and GSE138794) were included in this study. The intersection genes were obtained by overlapping differentially expressed genes (DEGs) from differential expression analysis in GSE16011, key module genes from WGCNA, and MRGs. Subsequently, the intersection genes were further screened to obtain prognostic genes. Following this, a risk model was developed and verified. After that, independent prognostic factors were identified, followed by the construction of a nomogram and subsequent evaluation of its predictive ability. Furthermore, immune microenvironment analysis and expression validation were implemented. The GSE138794 dataset was utilized to evaluate the expression of prognostic genes at a cellular level, followed by conducting an analysis on cell‐to‐cell communication. Finally, the results were validated in different datasets and tissue samples from patients.

**Results:**

ECI2, MCCC2, OXCT1, SUCLG2, and CPT2 were identified as prognostic genes for glioma. The risk model constructed based on these genes in TCGA‐GBMLGG demonstrated certain accuracy in predicting the occurrence of glioma. Additionally, the nomogram constructed based on risk score and grade exhibited strong performance in predicting patient survival. Significant differences were observed in the proportion of 27 immune cell types (e.g., activated B cells and macrophages) and the expression of 32 immune checkpoints (e.g., CD70, CD200, and CD48) between the two risk groups. Single‐cell RNA sequencing showed that CPT2, ECI2, and SUCLG2 were highly expressed in oligodendrocytes, neural progenitor cells, and BMDMs, respectively. The results of cell–cell communication analysis revealed that both oligodendrocytes and BMDMs exhibited a substantial number of interactions with high strength.

**Conclusion:**

This study revealed five genes associated with the prognosis of glioma (ECI2, MCCC2, OXCT1, SUCLG2, and CPT2), providing novel insights into individualized treatment and prognosis.

## Graphical abstract

This study constructed a prognostic model for mitochondria and macrophage polarization correlation in glioma based on single‐cell and transcriptome sequencing. Five genes associated with the prognosis of glioma (ECI2, MCCC2, OXCT1, SUCLG2, and CPT2) were revealed and then, validated using glioma tissues, providing novel insights into individualized treatment and prognosis.

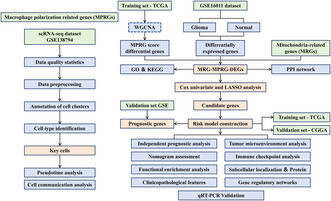

## 1 Introduction

Neuroglioma stands out as the prevailing primary tumor found within the central nervous system. The extreme proliferation and aggressiveness of neuroglioma cells contribute to a poor prognosis [1]. Based on the 2021 Classification of Central Nervous System Tumors by the World Health Organization, glioma is classified as grades I–IV, with patients with grades III and IV disease having a median survival of < 7.5 months. The incidence of glioma in adults is approximately 1 per 10,000 individuals [2]. Various approaches are currently employed for managing glioma, encompassing surgical excision, radiation therapy, pharmacotherapy, and immunomodulation; however, none of these methods are effective in controlling the recurrence of glioma. Given that the mechanisms involved in the development and progression of glioma remain elusive, potential prognostic markers should be identified and an in‐depth investigation into the pathological mechanisms of glioma should be conducted to improve prognosis.

Mitochondria, cytoplasmic organelles that are inherited maternally and originate from symbiotic bacteria, play a crucial role in cellular metabolism. In 1956, Otto Warburg discovered that cancer cells exhibited aerobic glycolysis, demonstrating that defective mitochondrial respiration plays a crucial part in the development of cancer [3]. Macrophage polarization is a dynamically balanced process of interconversion between M1 and M2 phenotypes. M1 macrophages can coordinate with other immune cells, such as T cells and natural killer cells, to eliminate antigens, thereby exerting pro‐inflammatory and anticancer effects. On the contrary, M2 macrophages can remodel damaged tissues, generate blood vessels, and participate in tissue repair, thereby causing immunosuppression, alleviating inflammation, and promoting cancer development [4]. Macrophage polarization is closely associated with cellular metabolism, and mitochondria play an important role in energy production and immunomodulation. The complex communication between macrophages and mitochondria, which involves ROS, glycolysis, and the TCA cycle, is tightly regulated during the development of glioma [5]. However, the underlying regulatory mechanisms remain unclear, necessitating further investigation.

Single‐cell RNA sequencing (scRNA‐seq) is a technique used to characterize the transcriptomic profile of each cell in a sample. It enables the identification of different cell populations and reveals commonalities and specificities in transcriptomic data and correlations within complex gene networks [6]. In cancer‐related research, different computational methods can be used to determine the relatively active signaling pathways in tumor and normal tissues, the regulatory networks among oncogenes or tumor suppressor genes, and the interactions between ligands and receptors, which can subsequently guide the treatment of cancer [7]. In this study, the prognostic significance of mitochondria‐related genes (MRGs) and macrophage polarization‐related genes (MPRGs) was assessed based on scRNAseq data extracted from glioma‐related public databases. Subsequently, cell–cell communication analysis was performed and the expression of relevant prognostic genes was evaluated at the single‐cell level in glioma. In addition, the potential regulatory mechanisms of the prognostic genes were examined, which provided a theoretical reference for the development of novel drugs and therapeutic strategies for glioma in clinical settings.

## 2 Materials and Methods

### 2.1 Data Acquisition

The University of California Santa Cruz (UCSC) (http://genome.ucsc.edu) database were applied to provide the glioma‐related dataset TCGA‐GBMLGG as training set 1, which contained the mRNA‐seq data and clinical information of 702 patients with low‐grade glioma (LGG) and glioblastoma multiforme (GBM), including 638 samples with survival information for subsequent analysis. The mRNA‐seq‐325 dataset (consisting of 182 cases in LGG and 139 cases in GBM) and the mRNA‐seq‐693 dataset (comprising of 443 cases in LGG and 249 cases in GBM) were obtained from Chinese Glioma Genome Atlas (CGGA) (http://www.cgga.org.cn) database as a validation set 1, resulting in 1013 glioma samples with RNAseq data and survival information. The GSE16011 (training set 2), GSE4290 (validation set 2), and GSE138794 (single‐cell dataset) datasets were obtained from the Gene Expression Omnibus (GEO) database (https://www.ncbi.nlm.nih.gov/). The GSE16011 dataset (platform: GPL8542) comprised 276 glioma samples and eight normal control samples, whereas the GSE4290 dataset (platform: GPL570) comprised 157 glioma samples and 23 normal control samples. The GSE138794 dataset (platform: GPL24676) comprised 28 brain tissue samples; of which, seven samples were included in subsequent analysis. Additionally, 1136 mitochondria‐related genes (MRGs) were obtained from the MitoCarta3.0 database (https://www.broadinstitute.org/mitocarta), and 35 macrophage polarization‐related genes (MPRGs) were obtained from published literature [8].

### 2.2 Differential Expression Analysis

In the GSE16011 dataset, differential expression analysis (glioma samples vs. control samples) was performed with limma package (v 3.58.1) [9] to obtain differentially expressed genes (DEGs), with the screening criteria set as false discovery rates (FDRs) of < 0.05 and |log2FoldChange (FC)| values of > 1. The DEGs were visualized on volcano maps generated using the ggplot2 package (v 3.4.4) [10], whereas their expression patterns were visualized on heat maps generated using the ComplexHeatmap package (v 2.16.0) [11].

### 2.3 Weighted Gene Co‐Expression Network Analysis

Weighted gene co‐expression network analysis (WGCNA) was implemented by the WGCNA package (v 1.72–5) [12] to identify genes associated with MPRGs scores in TCGA‐GBMLGG. Initially, cluster analysis was implemented on 638 glioma samples containing survival information to identify outliers. To guarantee the adherence of inter‐gene interactions to the scale‐free distribution, the optimal soft threshold (*β*) was determined. The adjacency of gene pairs and gene similarity based on adjacency were calculated. The dissimilarity coefficient between genes was derived, and a phylogenetic tree was subsequently constructed. According to the criteria set by the hybrid dynamic tree‐cutting algorithm, each gene module must contain a minimum of 400 genes in order to cluster them into distinct modules. The MEDissThres value of 0.4 was utilized to combine comparable modules that were examined using the dynamic tree‐cut algorithm in order to minimize redundancy.

Based on 35 MPRGs, the single‐sample gene set enrichment analysis (ssGSEA) algorithm of the GSVA package (v 1.50.0) [13] was utilized to reveal the score for the samples in TCGA‐GBMLGG. The optimal threshold for this score was calculated using the surv cutpoint function, and patients were divided into high and low‐score groups based on this threshold. Kaplan–Meier (K‐M) analysis was achieved through the survminer package (v 0.4.9) [14] to compare the survival rates of the two score groups (*p* < 0.05). Subsequently, the corrplot package (v 0.92) [15] was used to implement Spearman correlation analysis to identify modules exhibiting the most significant correlation with MPRG scores (cor > 0.3, *p* < 0.05). Genes in these modules were defined as key module genes for subsequent analysis.

### 2.4 Recognition of Candidate Genes

The intersection genes were obtained by intersecting DEGs, key module genes, and MRGs. The ggVennDiagram package (v 1.2.3) [16] to draw a Venn diagram to visualize this result. In order to further study the biological pathways and functions of the intersection genes, Gene Ontology (GO) and Kyoto Encyclopedia of Genes and Genomes (KEGG) enrichment analyses were implemented through clusterProfile package (v 4.10.0) [17] (*p*.adjust < 0.05). Furthermore, to explore whether there were interactions between intersection genes, the construction of protein–protein interaction (PPI) network was developed based on the STRING (http://string.embl.de/) database (confidence = 0.4). This network was visualized using Cytoscape software (v 3.10.0) [18]. Following this, the MCODE plug‐in in Cytoscape was utilized for cluster analysis of significant modules based on the relationship between edges and nodes in the PPI network. The initial screening criteria consisted of a *K*‐core value of 2, a degree cutoff of 2, a maximum depth limit of 100, and a node score threshold of 0.2. Candidate genes were identified from the most significant module obtained through clustering.

### 2.5 Screening of Prognostic Genes in Glioma

Candidate genes were sequentially included in univariate Cox regression analysis ((HR) ≠ 1 and *p* < 0.05), proportional hazards (PH) hypothesis testing (*p* > 0.05), and least absolute shrinkage and selection operator (LASSO) regression analysis to obtain prognostic genes. Specially, the glmnet package (v 4.1–8) [19] was utilized to conduct LASSO analysis. The genes identified via LASSO analysis were defined as prognostic genes.

### 2.6 Construction and Evaluation of a Risk Model

Based on the prognostic genes obtained from the above analysis, a risk model was constructed. The riskScore was calculated as: riskRcore = $\sum_{i=1}^{n}\left(\beta_{i}\times {\text{Expr}}_{i}\right)$. In this equation, the LASSO coefficient of prognostic gene *i* was denoted as *β*_*i*_, while Expri represented the relative expression level of prognostic gene *i*. The surv cutpoint function was employed to determine the optimal threshold for the risk score. Subsequently, patients in the TCGA‐GBMLGG dataset were stratified into two risk subgroups (high and low‐risk groups) based on this threshold. Scatter plots were generated to assess the survival status of patients within different risk subgroups, whereas heat maps were generated to evaluate the expression of prognostic genes across distinct risk subgroups. After that, the K‐M survival curve was plotted via survminer package (v 0.4.9) [20] to compare survival differences between these risk subgroups. Meanwhile, the timeROC package (v 0.4) [21] was employed to construct receiver operating characteristic (ROC) curves to evaluate the predictive accuracy of the risk model. The analytical approach was employed to validate the risk model in validation set 1. Furthermore, patients in the CGGA database were categorized into high‐risk and low‐risk groups based on the optimal thresholds for risk scores, and K‐M survival curves were also plotted by the survminer software package (v 0.4.9) [20] to compare the survival differences between these risk subgroups for further validation.

### 2.7 Construction and Evaluation of a Nomogram

In the TCGA‐GBMLGG dataset, a comprehensive analysis was implemented to identify independent prognostic factors by integrating the risk score and four clinical features. This involved performing univariate Cox regression analysis (*p* < 0.05), pH hypothesis testing (*p* > 0.05), and multivariate Cox regression analysis (*p* < 0.05). Subsequently, based on the identified independent prognostic factors, the rms package (v 6.7–0) [22] was employed to construct a nomogram to analyze the possible 1‐, 3‐, and 5‐year survival rates of patients with glioma. Furthermore, calibration curves and decision curve analysis (DCA) were created to reveal the predictive power of the nomogram for patients with gliomas. Additionally, the Wilcoxon test was utilized to examine the variations in riskScore between the subgroups of four clinical features, as well as to assess disparities in prognostic genes across these subgroups. A violin plot was generated for the visual representation of the results.

### 2.8 Gene Set Enrichment Analysis (GSEA)

In order to comprehend the disparities in biological function between glioma patients belonging to two risk subgroups, differential expression analysis was implemented in TCGA‐GBMLGG dataset via DEseq2 package (v 1.42.0) [23] based on c2.cp.kegg.v2023.1.Hs.symbols.gmt as reference gene set, which obtained from Molecular Signatures Database (MSigDB) (https://www.gsea‐msigdb.org/gsea/msigdb). The log2FC was calculated and arranged in descending order of magnitude. Subsequently, GSEA was implemented by clusterProfiler package (v 4.10.0).

### 2.9 Immune Microenvironment Analysis

The infiltration scores of 28 immune cell types in two risk subgroups were calculated using the ssGSEA algorithm of GSVA package (version 1.44.5). A heat map was generated to show the infiltration scores of 28 immune cells. Subsequently, the Wilcoxon test was implemented to assess the disparity in the infiltration of 28 immune cell in the two risk subgroups. Spearman correlation analysis was conducted using Corrplot package (v 0.92) to examine the relationship between differential immune cells and prognostic genes, followed by the generation of a heat map to visually present the findings (*p* < 0.05). In addition, the comparison of immune checkpoints [24] expression was achieved between the two risk subgroups, and the correlation between prognostic genes and immune checkpoints was examined.

### 2.10 Subcellular Localization

Given that the functions of genes are closely related to their locations, we analyzed the subcellular localization of prognostic genes using the mRNA Locater tool (http://bio‐bigdatacn/mRNALocater).

### 2.11 Construction of Regulatory Networks

Regulatory networks were constructed to investigate molecular mechanisms included in the regulation of prognostic genes in glioma. The miRWalk (http://mirwalk.umm.uni‐heidelberg.de/search_genes/) and Starbase (http://starbase.sysu.edu.cn/in) databases were employed to predict miRNAs regulating prognostic genes. The miRNAs obtained from both databases were intersected to identify key miRNAs. Subsequently, the Starbase and miRNet (http://xeno.mirnet.ca.) databases were utilized to predict upstream lncRNAs associated with key miRNAs. The lncRNAs obtained from both databases were intersected to identify key lncRNAs. The prognostic genes, key miRNAs, and key lncRNAs were integrated to construct an lncRNA–miRNA–mRNA network. Additionally, the hTFtarget (http://bioinfo.life.hust.edu.cn/hTFtarget#!/) database was employed to predict transcription factors (TFs) targeting prognostic genes, and a TF–mRNA network was subsequently constructed.

### 2.12 Single‐Cell RNA‐Seq

The single‐cell RNA‐seq analysis was implemented to investigate the expression of prognostic genes at the cellular level. Ineligible samples were removed through quality‐control analysis, and the Seurat package (v 4.4.0) [25] was used to create a Seurat object (min.cells = 3; min.features = 200). Subsequently, the scDblFinder package (v 1.16.0) [26] was used to perform two‐cell detection to identify cells exhibiting mitochondrial body content below 10%. The FindVariableFeatures function was used to identify genes with high variability after quality‐control analysis. Based on the highly variable genes, principal component analysis (PCA) was performed to assess cell distribution and identify outliers. The percentage of variance (information) represented by each principal component (PC) was evaluated, and a PCA inflection point map was plotted. PCs in front of the inflection point were selected for subsequent analysis. The Seurat package (v 4.4.0) was utilized for unsupervised clustering of filtered cells, and the FindNeighbors and FindClusters functions were employed for dimensionality reduction. The results were visualized through the uniform manifold approximation and projection (UMAP) algorithm. Furthermore, the clustered cells were annotated based on marker genes mentioned in a previous study [27] to determine the cell type. Dot plots were generated to demonstrate the expression of marker genes in different cell types. Additionally, the expression of prognostic genes was examined in different cell types, and cells exhibiting higher expression were identified as key cells.

### 2.13 Pseudo‐Time and Cell–Cell Communication Analyses

Pseudo‐time analysis was performed based on key cells. Single‐cell trajectory maps were constructed using the Monocle 2 algorithm to project all cells in a single‐cell cluster onto a root and two branches. Subsequently, the expression of prognostic genes was evaluated along different time points. The CellChat tool was used to quantify receptor–ligand interactions across different cell types, enabling the inference of intercellular communication networks. The pheatmap package (v 1.0.12) [28] was utilized to generate heat maps to visualize interaction pathways specific to each cell type.

### 2.14 Validation of the Expression of Prognostic Genes

The expression levels of prognostic genes in glioma and control samples from both GSE16011 and GSE4290 datasets were compared using the Wilcoxon test. In addition, a comparison was made between tumor and normal tissues in the HPA database to assess the protein expression of these prognostic genes. Finally, qPCR was implemented to validate the expression of prognostic genes in cancer and para‐cancer tissues from patients with glioma.

### 2.15 Statistical Analysis

The data were processed and analyzed using the R software (version 4.2.1). To assess differences between groups, the Wilcoxon test was employed. Statistical significance was determined at a *p* value below 0.05.

## 3 Results

### 3.1 A Sum of 5107 DEGs and 4593 Key Module Genes Were Identified on Glioma

**FIGURE 1:**
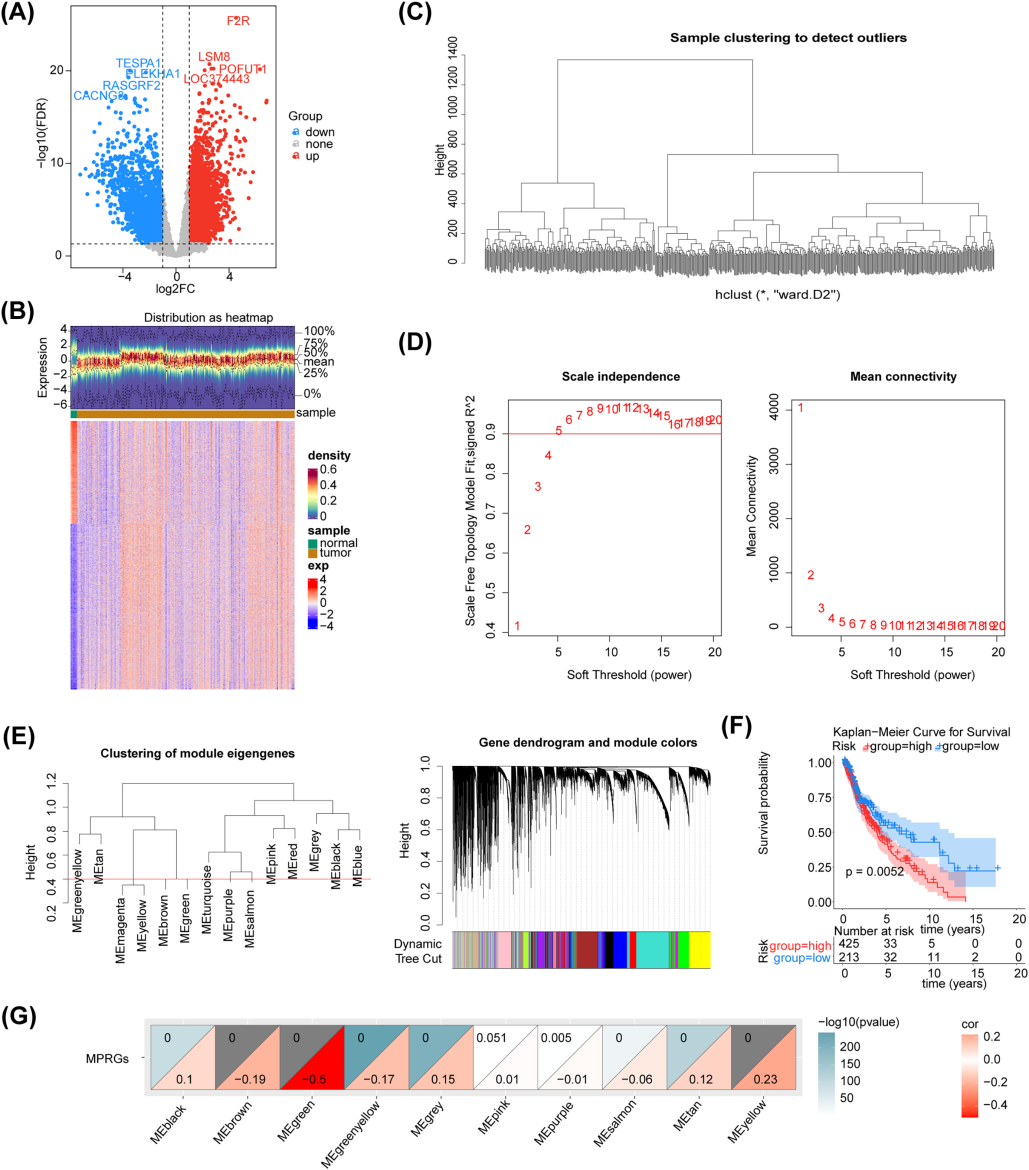
Screening of DEGs and key module genes. (A) Volcano map of DEGs between glioma and control groups in GSE16011 dataset. (B) Heat map of DEGs between glioma and control groups in GSE16011 dataset. (C) Clustering of samples in WGCNA. (D) Scale‐free soft threshold distribution. (E) Tree diagram of co‐expression network modules. (F) K‐M survival analysis between high and low expression groups of MPRGs score. (G) Correlation heat map between modules and MPRGs score.

Based on differential expression analysis, 5107 DEGs were identified for glioma in the GSE16011 dataset. Of these DEGs, 3145 were up‐regulated while 1962 were down‐regulated (Figure 1A,B). WGCNA was implemented to identify genes associated with MPRG scores in training set 1. Cluster analysis revealed no outliers in the samples (Figure 1C). As shown in Figure 1D, the optimal soft thresholding value (*β*) was estimated to be five when the scale‐free R2 on the ordinate exceeded the threshold of 0.9 (represented by a red line) in conjunction with a decrease in mean connectivity toward zero in the graph on the right side. A total of 10 modules were obtained after similar modules were merged (Figure 1E).

The K‐M survival analysis revealed a significant disparity in survival rates between the two score groups, based on the optimal threshold (6.148717) of ssGSEA score for MPRGs (Figure 1F). The obtained result suggested the feasibility of utilizing this score as a trait for identifying gene modules that exhibited significant associations with it. Furthermore, correlation analysis demonstrated that MEgreen exhibited the most significant negative correlation with ssGSEA scores (cor = −0.5, *p* < 0.05), indicating a strong association among the genes in this module with MPRGs (Figure 1G). Therefore, this module was identified as a key module, encompassing 4593 genes.

### 3.2 Identification of Five Candidate Genes for Glioma

**FIGURE 2:**
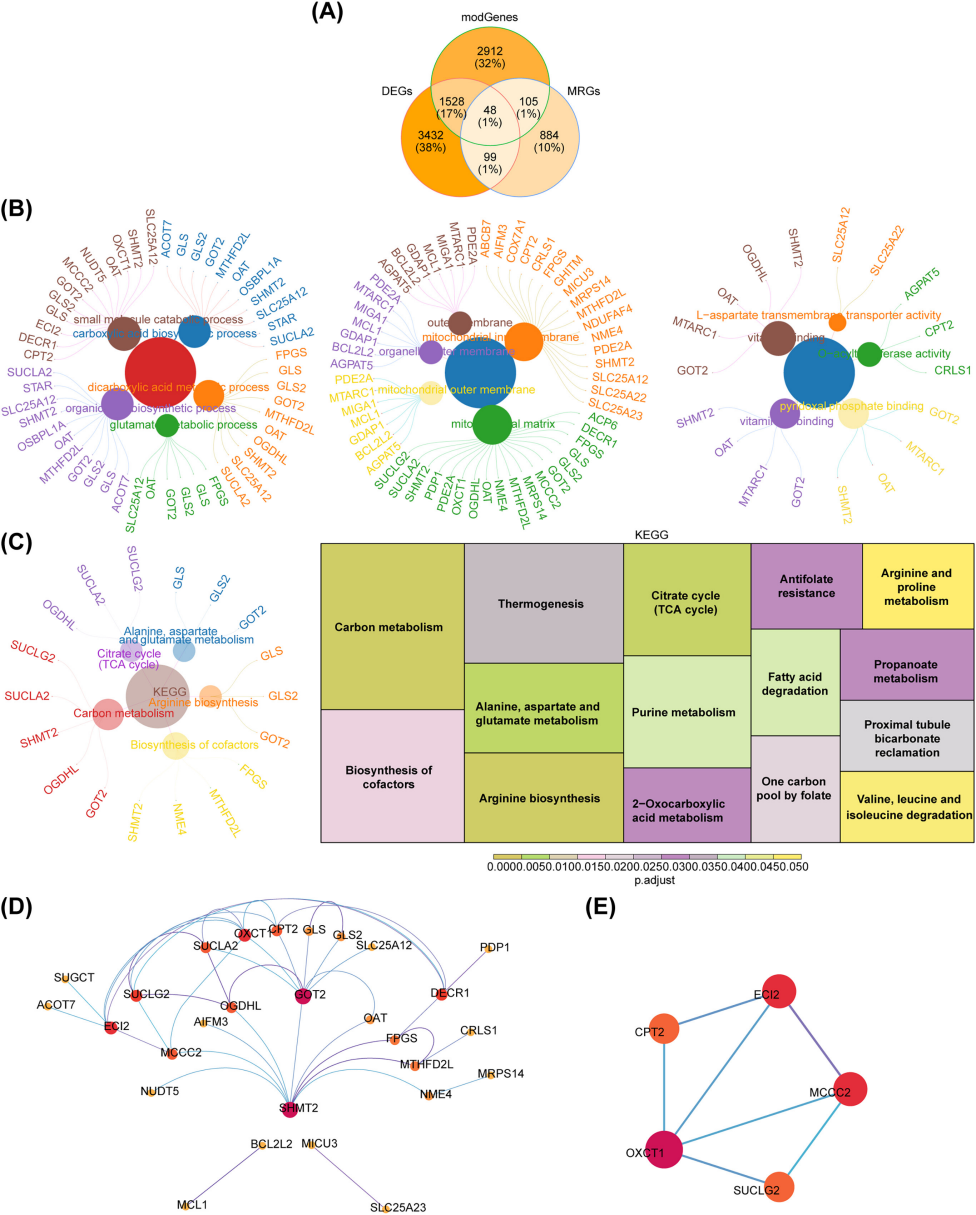
Identification of candidate genes. (A) Venn diagrams for intersection genes. (B) GO enrichment plot of intersection genes. (C) KEGG enrichment plot of intersection genes. (D) PPI networks for intersection genes. (E) Identification of candidate genes.

The intersection of 5107 DEGs; 4593 key module genes; and 1136 MRGs revealed 48 overlapping genes (Figure 2A). These overlapping genes were enriched in 149 GO terms and 15 KEGG pathways. The GO terms consisted of 10 cellular components (CCs); 24 molecular functions (MFs); and 115 biological processes (BPs), such as mitochondrial matrix, small‐molecule catabolic process, and glutamate metabolic process (Figure 2B). The enriched KEGG pathways included carbon metabolism, arginine biosynthesis, biosynthesis of cofactors, and citrate cycle (TCA cycle) (Figure 2C). Furthermore, the PPI network revealed interactions between 28 overlapping genes (Figure 2D); among which, ECI2, MCCC2, OXCT1, SUCLG2, and CPT2 were identified as candidate genes (Figure 2E).

### 3.3 The 5‐Gene Risk Model Effectively Predicted the Prognosis of Glioma

**FIGURE 3:**
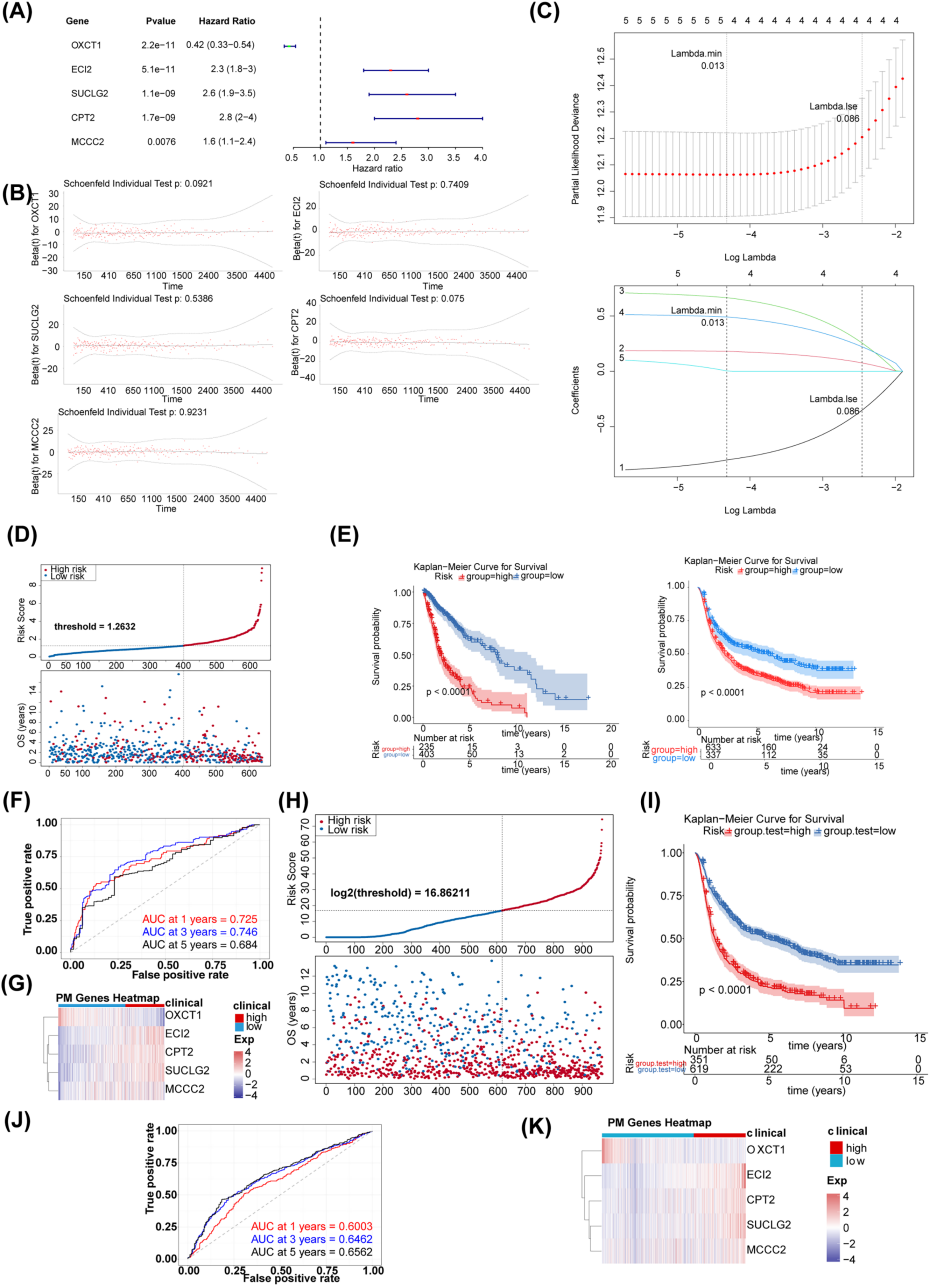
Construction and evaluation of risk model. (A) Univariate Cox regression analysis forest plot. (B) PH hypothesis test. (C) LASSO regression analysis. (D) Survival scatterplot in TCGA‐GBMLGG dataset. (E) K‐M survival curves. The left panel shows the K‐M survival curves for the TCGA dataset. The right panel shows the K‐M survival curves for the CGGA dataset. (F) ROC curves in TCGA‐GBMLGG dataset. (G) Expression of prognostic genes in TCGA‐GBMLGG dataset. (H) Survival scatterplot in validation set 1. (I) KM survival curves in the validation set 1. (J) ROC curves in the validation set 1. (K) Expression of prognostic genes in the validation set 1.

After conducting univariate Cox regression analysis (Figure 3A), PH hypothesis testing (Figure 3B), and LASSO regression analysis (Figure 3C), five prognostic genes were identified, including ECI2, MCCC2, OXCT1, SUCLG2, and CPT2. Based on these prognostic genes, a risk model was constructed. The risk score was calculated as follows: (−0.9201) × OXCT1 + 0.1900 × ECI2 + 0.7230 × SUCLG2 + 0.5188 × CPT2 + 0.1340 × MCCC2. As shown in the scatter plot in (Figure 3D), the K‐M survival curve for the TCGA dataset and K‐M survival curves for the CGGA dataset (Figure 3E), patients in the high‐risk group had significantly shorter OS. The AUC values achieved high accuracy in prognosticating glioma outcomes, with scores of 0.725, 0.746, and 0.684 at the respective time points of 1 year, 3 years, and 5 years (Figure 3F). The expression of prognostic genes in the two risk groups is shown in Figure 3G. Furthermore, the predictive accuracy of the risk model was evaluated in validation set 1. The scatter plot (Figure 3H), K‐M curve (Figure 3I), and ROC curve (Figure 3J) were consistent with those in training set 1, suggesting that the risk model was effective in predicting prognosis. The expression of prognostic genes in different groups in validation set 1 is shown in Figure 3K.

### 3.4 A Nomogram Based on Independent Prognostic Factors Effectively Predicted the Prognosis of Glioma

**FIGURE 4:**
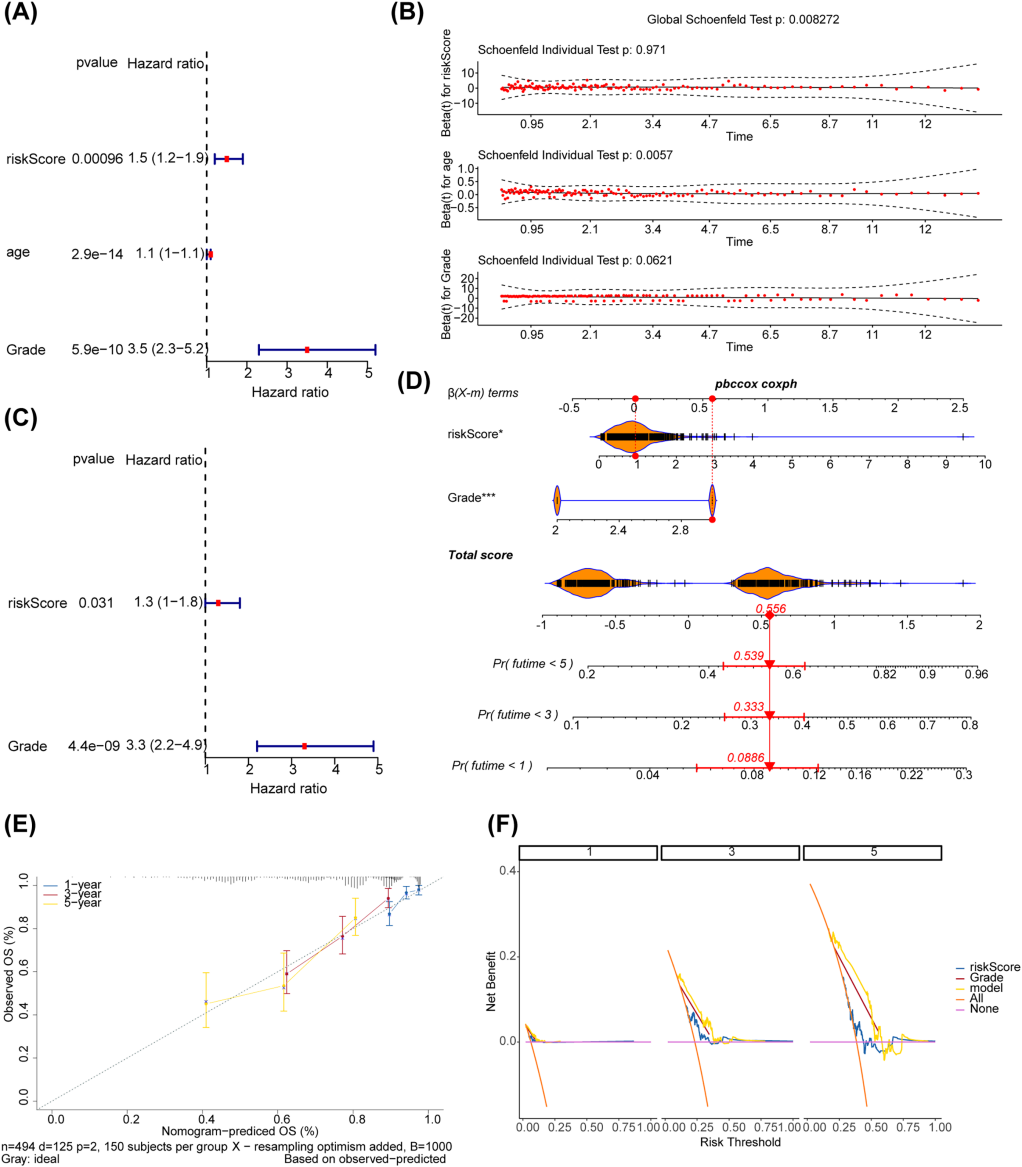
Creation and evaluation of nomogram. (A) Univariate Cox regression analysis forest plot. (B) pH hypothesis test. (C) Multivariate Cox regression forest plot. (D) Nomogram to predict patient survival at 1, 3, 5 years. (E) Calibration curve of the nomogram. (F) DCA of the nomogram.

Through a series of screening, the risk score and tumor grade were independent prognostic factors (Figure 4A–C). A nomogram was developed utilizing various independent prognostic factors to estimate the survival outcome for individuals diagnosed with glioma (Figure 4D). The slope of the calibration curve of the nomogram was close to 1 (Figure 4E), and the DCA curve of the nomogram was above the other curves (Figure 4F), indicating that the nomogram model had certain predictive accuracy. Furthermore, the risk score was compared between subgroups stratified based on distinct clinical features. The results indicated that the risk score was significantly associated with only grade, with the G3 group having a higher risk score (Figure S1). Similarly, the expression of prognostic genes was significantly different only between the subgroups stratified based on grade (Figure S2).

### 3.5 Functional Pathways and Immune Analysis of the Two Risk Groups

**FIGURE 5:**
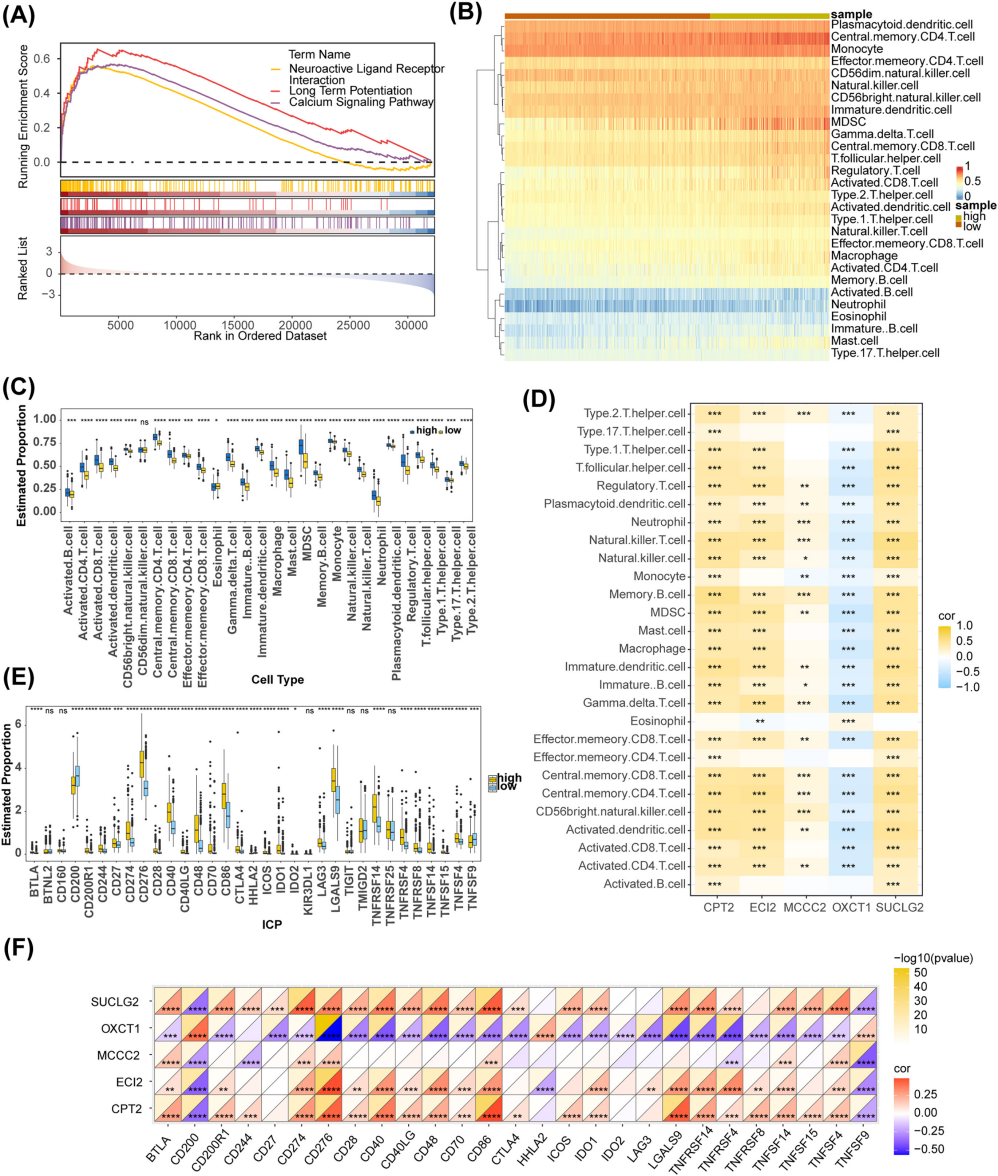
Functional pathways and immune analysis. (A) GSEA enrichment result. (B) Heat map of the immune cell infiltration level between the two risk subgroups. (C) Identification of differential immune cells. (D) Heat map of correlation between prognostic genes and differential immune cells. (E) Differences in expression of immune checkpoints between the two risk subgroups. (F) Heatmap of correlation between prognostic genes and differential immune checkpoints.

GSEA demonstrated that 36 pathways were enriched, of which the pathways related to glioma were calcium signaling pathway, neuroactive ligand–receptor interaction, hematopoietic cell lineage, cytokine–cytokine receptor interaction, ECM–receptor interaction and so on (Table S1). The top three pathways are shown in Figure 5A,B displayed the levels of immune cell infiltration in the two risk subgroups. The infiltration levels of all immune cell types except CD56dim natural killer cells were significantly different between the two risk groups. All immune cells except eosinophils exhibited a higher infiltration level in the high‐risk group (Figure 5C). Correlation analysis showed that the expression of ECI2, MCCC2, SUCLG2, and CPT2 was significantly positively correlated with the infiltration levels of most differential immune cells, whereas the expression of OXCT1 showed the opposite trend (Figure 5D). Specially, the most significant positive correlation with natural killer T cell was observed for SUCLG2 (cor = 0.55, *p* < 0.05), whereas OXCT1 exhibited the most significant negative correlation with gamma delta T cell (cor = −0.47, *p* < 0.05) (Table S2). Furthermore, the expression of 32 immune checkpoints was significantly different between the two risk subgroups. Specifically, the expression of all immune checkpoints except CD200, HHLA2, and TNFSF9 was significantly higher in the high‐risk group (Figure 5E). OXCT1 exhibited a significant negative correlation with most differentially expressed immune checkpoints but a significant positive correlation with CD200, HHLA2, and TNFSF9. Similarly, SUCLG2, ECI2, and CPT2 exhibited a significant positive correlation with most differentially expressed immune checkpoints but a negative correlation with CD200 and TNFSF9 (Figure 5F).

### 3.6 Analysis of Protein Expression and Regulatory Mechanisms of Prognostic Genes

**FIGURE 6:**
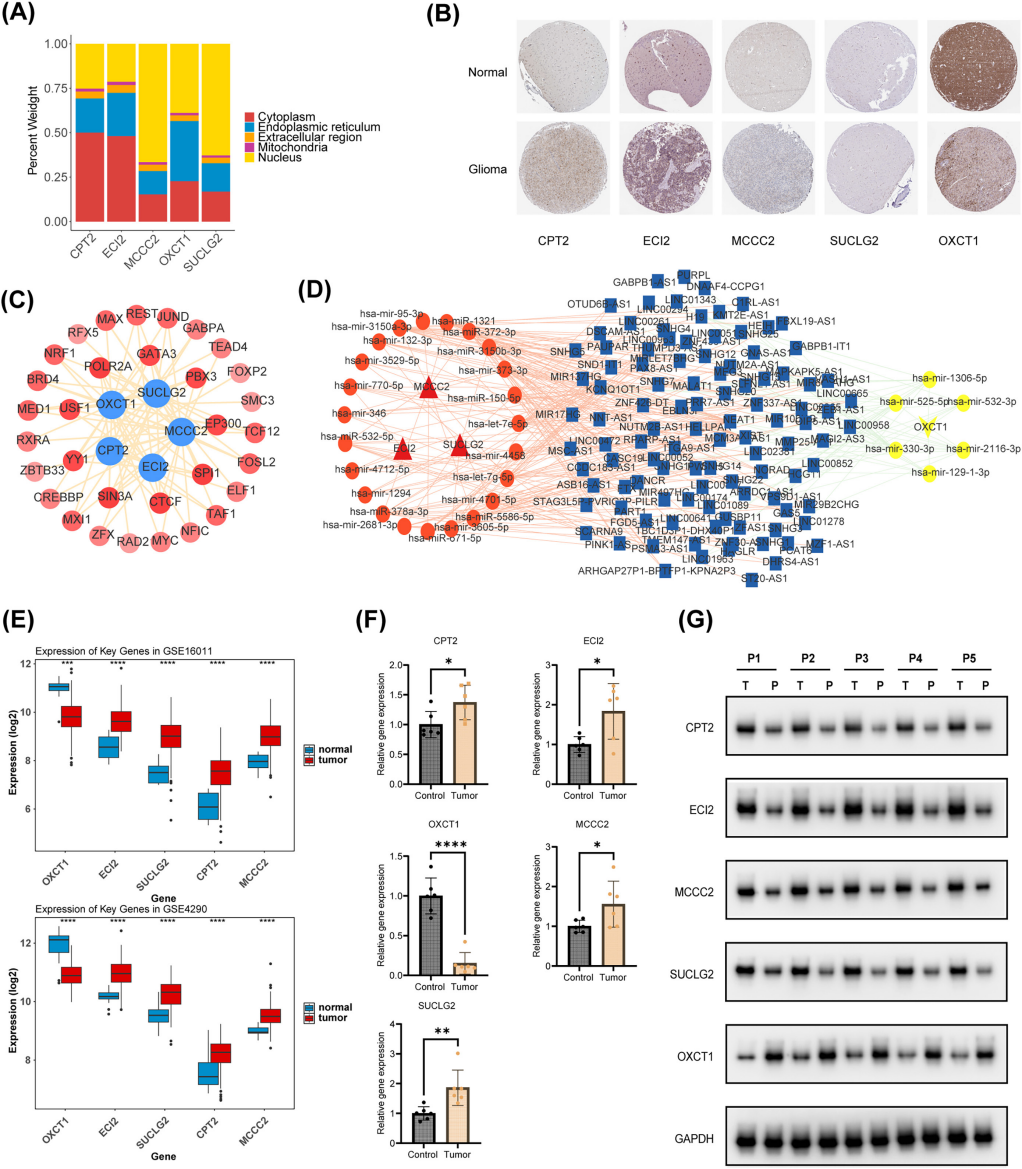
Analysis of protein expression, regulatory mechanisms and validation of prognostic genes. (A) Subcellular localization of prognostic genes. (B) The protein expression of prognostic genes between glioma and control samples from the HPA database. (C) TF‐mRNA network. (D) LncRNA‐miRNA‐mRNA network. (E) Expression levels of prognostic genes in glioma and normal controls in GSE16011 and GSE4290 datasets. (F) and (G) The qPCR and WB results of prognostic genes in glioma tissues and paracancerous tissues.

Subcellular localization analysis demonstrated that CPT2 and ECI2 were located mainly in the cytoplasm, whereas MCCC2, OXCT1, and SUCLG2 were located mainly in the nucleus (Figure 6A). The protein expression of the five prognostic genes in glioma and control samples is shown in (Figure 6B). The key miRNAs and lncRNAs regulating the prognostic genes are listed in (Table S3) The lncRNA–miRNA–mRNA network revealed that XIST regulated MCCC2 through hsa‐mir‐3150a‐3p, hsa‐miR‐1321, hsa‐miR‐372‐3p, hsa‐mir‐373‐3p, and hsa‐let‐7e‐5p; SUCLG2 through hsa‐miR‐3150b‐3p, hsa‐miR‐150‐5p, hsa‐mir‐4458, and hsa‐mir‐132‐3p; and OXCT1 through hsa‐mir‐330‐3p, hsa‐mir‐525‐5p, and hsa‐mir‐129‐1‐3p (Figure 6C). Furthermore, a total of 9, 26, 25, 20, and 11 TFs targeting ECI2, MCCC2, OXCT1, SUCLG2, and CPT2 were predicted, respectively. Among these TFs, CTCF, POLR2A, SIN3A, and SPI1 concurrently regulated all five prognostic genes in glioma (Figure 6D).

### 3.7 Totally Three Key Cells Were Identified Through Single‐Cell RNA‐Seq Analysis

**FIGURE 7:**
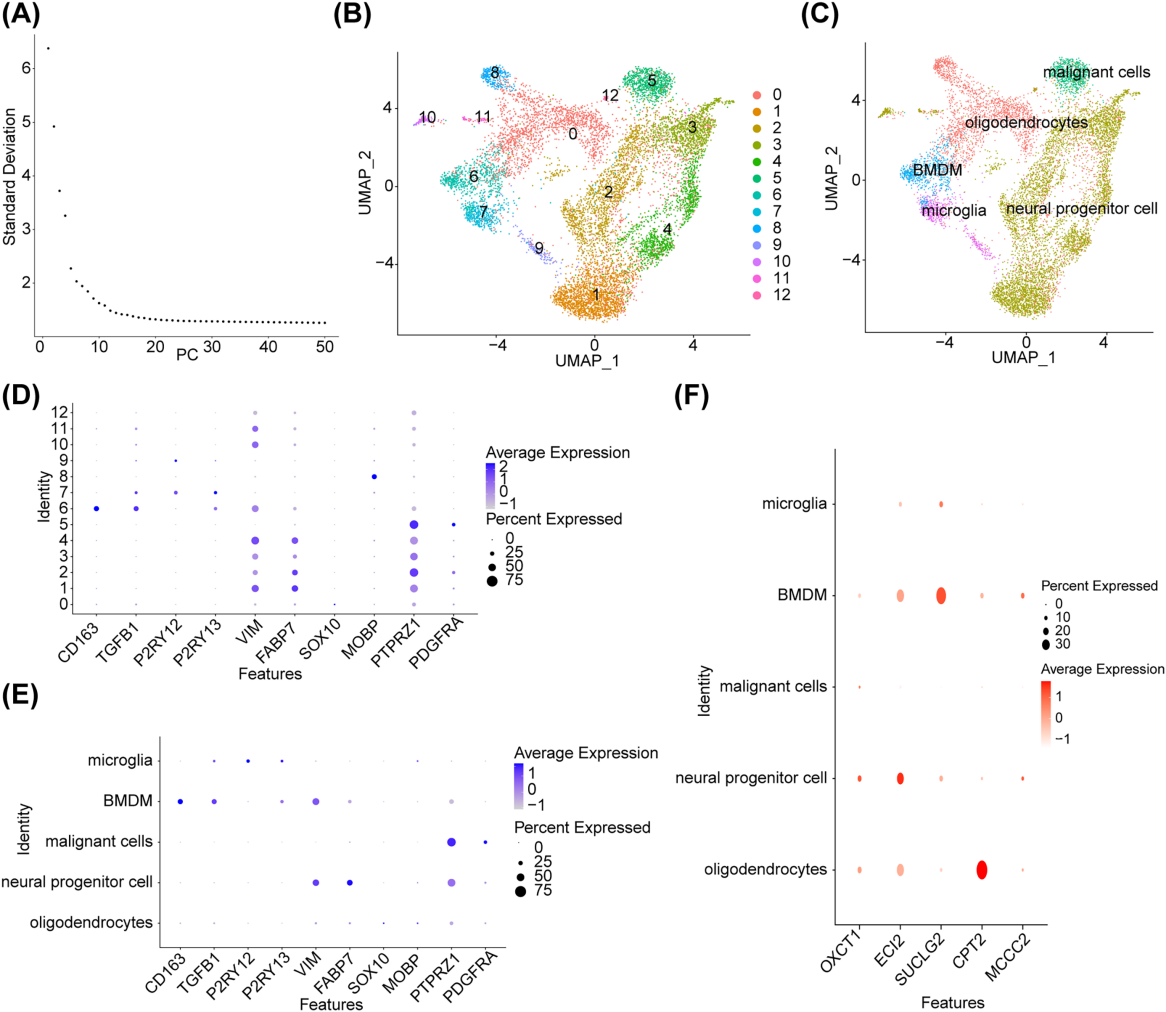
Identification of three key cells through single‐cell RNA‐seq analysis. (A) Plot of PCA inflection points. (B) UMAP cell clustering map. (C) Clustering map after cell annotation. (D) Prognostic gene expression in different cells. (E) Scatterplot of clustered marker genes expression before cell annotation. (F) Scatterplot of clustered marker genes expression after cell annotation.

The cell types included in the quality control analysis are shown in Figure S3. After quality control, a total of 10,701 cells were retained (Figure S4). The top 2000 highly variable genes are shown in Figure S5. PCA revealed no evident outliers in the samples (Figure S6A), and the top 13 PCs were selected for subsequent analysis (Figure 7A). Dimensionality reduction in cluster analysis revealed 13 cell subsets (Figure 7B), which were subsequently categorized into five cell types, namely, oligodendrocytes, neural progenitor cells, malignant cells, bone marrow‐derived macrophages (BMDMs), and microglia (Figure 7C). The expression of marker genes in different cell types before and after an annotation is shown in Figure 7E,F, respectively. In particular, CPT2, ECI2, and SUCLG2 were highly expressed in oligodendrocytes, neural progenitor cells, and BMDMs, respectively. Therefore, these three cell types were selected as key cells (Figure 7D).

### 3.8 Analysis of Differentiation Trajectories and Interactions of Key Cells

**FIGURE 8:**
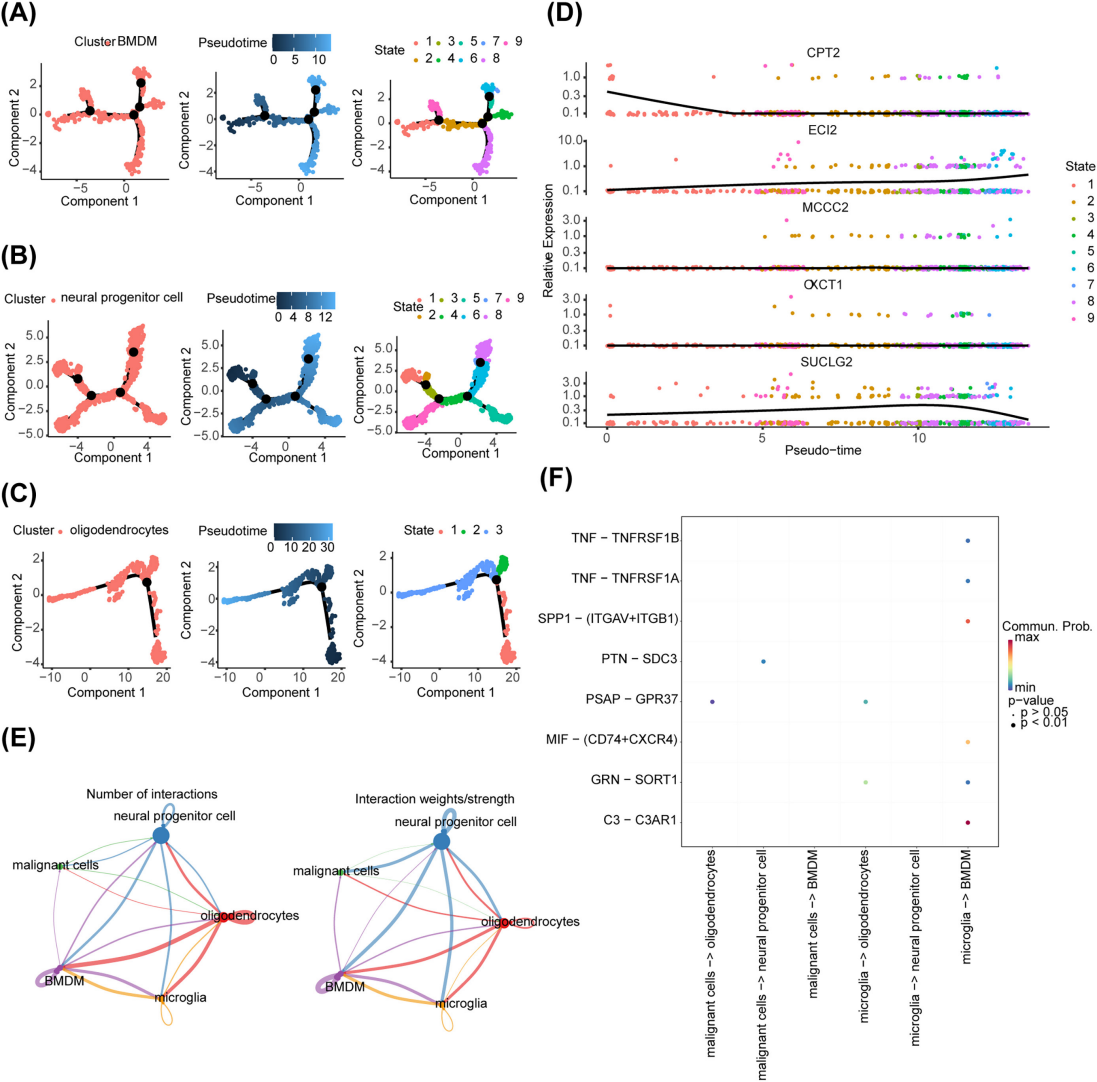
Analysis of differentiation trajectories and interactions of key cells. (A) The differentiation trajectories of BMDM. (B) The differentiation trajectories of neural progenitor cells. (C) The differentiation trajectories of oligodendrocytes. (D) Trends of prognostic gene expression at various stages with BMDM cell differentiation. (E) Cellular communication between the five cell types is obtained by annotation. (F) Interaction pathways for each cell type.

The differentiation trajectories of BMDMs, neural progenitor cells, and oligodendrocytes are shown (Figure 8A–C). The overall expression of OXCT1 and MCCC2 in BMDMs remained relatively stable during differentiation. The expression of CPT2 decreased during the late stage of state 1 and stabilized thereafter. The expression of ECI2 exhibited an overall increase during differentiation. The expression of SUCLG2 increased in the initial stage but decreased in the final stage of differentiation (Figure 8D). However, the expression of the five prognostic genes remained unaltered during the differentiation of neural progenitor cells and oligodendrocytes (Figure S6B–C). The results of cell–cell communication analysis of the five cell types showed that both oligodendrocytes and BMDMs exhibited a large number of interactions, with a high interaction strength. As shown in the image on the right side (Figure 8E), neural progenitor cells exhibited significantly strong interactions with the other four cell types, indicating their crucial role in cell–cell communication. The interaction pathways of each cell type are shown in Figure 8F.

### 3.9 Prognostic Genes Showed Consistent Expression Trends in Two Datasets

The expression of the five prognostic genes was significantly different between glioma and control samples in the GSE16011 and GSE4290 datasets, demonstrating consistency in both datasets (Figure 6E). In addition, qPCR and Western blot showed that the expression of the five prognostic genes was significantly different between cancer and para‐cancer tissues from patients with glioma (Figure 6F,G). Notably, the expression of ECI2, MCCC2, SUCLG2, and CPT2 was higher and that of OXCT1 was lower in cancer tissues than in para‐cancer tissues.

## 4 Discussion

In glioma, tumor proliferation, migration, and invasion are closely associated with cellular metabolism, anti‐tumor immunity, and other complex pathophysiological processes [1]. Macrophage polarization refers to the differentiation of macrophages into M1 and M2 subtypes with pro‐inflammatory and anti‐inflammatory functions, respectively. Mitochondria play a crucial role in cellular metabolism. In addition to energy production, several biocommunication activities involved in mitochondrial metabolism are closely associated with glioma and macrophage polarization [29]. In this study, we identified five prognostic genes associated with mitochondrial and macrophage polarization in glioma and constructed a risk model via bioinformatic analysis. The findings of this study not only provide a theoretical reference for investigating the mechanisms underlying mitochondrial and macrophage polarization in glioma but also offer valuable insights into the diagnosis, prognosis, and treatment of glioma.

A total of 48 overlapping genes were identified after intersecting 5107 DEGs; 4593 key module genes; and 1136 MRGs. GO analysis showed that the overlapping genes were primarily enriched in the mitochondrial matrix, mitochondrial inner membrane dicarboxylic acid (DCA) metabolism, small molecule catabolism, and glutamate metabolism. DCA is a product of fatty acid oxidation, particularly mitochondrial β‐oxidation. The metabolism of dicarboxylic acid is usually more active in the liver and kidney, which can effectively inhibit toxic reactions caused by fatty acid aggregation [30]. Glutamate, a key metabolite in the tricarboxylic acid (TCA) cycle and glycolysis that occurs in mitochondria, serves as an early indicator of the efficacy of the chemotherapeutic agent temozolomide in glioma. It has been shown to improve the prognosis of IDH1‐mutant glioma. Additionally, its metabolic mechanisms, processes, and related products are important for macrophage polarization [31, 32]. KEGG analysis revealed that the overlapping genes were enriched in pathways related to carbon metabolism, citrate cycle, arginine biosynthesis, and biosynthesis of cofactors. The development of glioma involves many complex metabolic pathways and products, such as the TCA cycle, glycolysis, and fatty acid metabolism [33]. Carbon metabolism primarily meets the energy requirements of healthy brain and tumor cells, more often transferring energy in the form of glucose. The TCA cycle provides a sufficient amount of ATP for glioma development and involves the complex biosynthesis process that metabolizes abundant neurotransmitters. In addition, various nitrogen substances, resembling a wide range of amino acids and nucleotides, can regulate mitochondrial metabolism through a series of biochemical pathways, such as deamination or transamination [34]. Therefore, the biological functions and pathways of the overlapping genes identified in this study play an integral role in mitochondrial metabolism, macrophage polarization, and glioma development.

The five prognostic genes identified in this study include ECI2, MCCC2, OXCT1, SUCLG2, and CPT2. 3‐oxoacid CoA‐transferase 1 (OXCT1) promotes glycolysis by catalyzing the breakdown of ketone bodies, thereby contributing to the proliferation of glioma cells. Therefore, targeting OXCT1 to increase ketone body levels to inhibit glycolysis represents a promising therapeutic strategy for glioma [35]. Enoyl‐CoA‐(Δ) isomerase 2 (ECI2) is involved in fat metabolism, which can promote the development of prostate cancer [36]. Methylcrotonoyl‐CoA carboxylase 2 (MCCC2), a mitochondria‐related gene that catabolizes leucine during metabolism, has been shown to have different expression patterns in breast and colorectal cancers [37]. Succinyl‐coenzyme A synthetase GDP‐forming subunit *β* (SUCLG2) not only participates in the TCA cycle but also maintains the stability of mitochondrial DNA. It is upregulated in tissues with higher metabolic levels in lung and prostate cancers [38]. Carnitine palmitoyltransferase 2 (CPT2) is highly expressed in recurrent GBM, and its involvement in mitochondrial fatty acid oxidation with macrophage‐driven phagocytosis of CD47 contributes to a poor prognosis [39]. The high expression of CPT2 in GBM has been associated with free fatty acids promoting tumor development [40]. To the best of our knowledge, this study is the first to demonstrate that ECI2, MCCC2, and SUCLG2 are promising prognostic biomarkers and therapeutic targets for glioma.

Furthermore, the top three signaling pathways significantly enriched in the high‐risk group included neuroactive ligand–receptor interaction, long‐term potentiation, and calcium signaling. When involved in ubiquitination, the Parkin receptor degrades misfolded proteins and regulates the glioma microenvironment [41]. The calcium signaling pathway is involved in metabolic programming and regulates the expression of cytoskeleton‐related proteins. Increased intracellular calcium levels can activate calcium signaling, promoting the proliferation and recurrence of glioma by facilitating glutamate release and synaptic [42]. Studies on animal models of glioma have demonstrated that adoptive cell therapy (ACT) can facilitate the transfer of hematopoietic stem and progenitor cells (HSPCs) from the bone marrow, thereby resulting in a good prognosis. Therefore, hematopoietic cells hold potential clinical value in the treatment of glioma [43]. Altogether, the five prognostic genes identified in this study may serve as valuable diagnostic biomarkers and therapeutic targets for glioma.

Immune infiltration analysis revealed that the proportion of 27 immune cell types was significantly different between the two risk subgroups. Among these cell types, tumor‐associated macrophages (TAMs) play a crucial role in the development of an immunosuppressive tumor microenvironment (TME). In glioma, various non‐coding RNAs can mediate the polarization of TAMs, thereby affecting immune regulation. For example, hsa_circ_0001460 (named circNEIL3) can induce macrophage polarization by inhibiting RNA‐binding proteins, subsequently modulating immune function in the TME. However, the mechanism underlying this phenotypic transformation remains unclear, and further investigation is warranted to develop immunotherapies for glioma in the future [44]. In the TME of glioma, myeloid‐derived suppressor cells (MDSCs) can inhibit T‐cell differentiation and immune gene expression to induce immunosuppression and promote tumor proliferation and invasion. miR‐1246 has been shown to activate MDCSs through the ERK signaling pathway, suggesting its potential as a novel target against MDSCs in glioma [45]. Furthermore, the expression of 32 immune checkpoints was significantly different between the high‐ and low‐risk groups. Peptide inhibitors targeting CD200 have been shown to enhance immune function in TME by modulating cytokines and dendritic cells, resulting in the suppression of glioma [46].

The results of scRNA‐seq revealed five cell types, namely, neural progenitor cells (NPCs), oligodendrocytes, bone marrow‐derived macrophages (BMDMs), malignant cells, and microglia. NPCs, derived from neural stem cells, are primitive and crucial components of the nervous system. Brain tumor stem cells (BTSCs) share biological characteristics and many migratory pathways with NPCs and have been associated with the degree of malignancy in glioma [47]. BTSCs are highly proliferative and resistant to radiotherapy and chemotherapy. Highly migratory NPCs can help these BTSCs invade and infiltrate brain tissues, which makes it difficult to control the development of glioma. In this study, cell–cell communication analysis showed that NPCs exhibited strong interactions with the remaining four cell types, suggesting their important role in intercellular communication in glioma. In addition, oligodendrocytes and BMDMs exhibited a high number and intensity of interactions. Previous studies have shown that oligodendrocyte precursor cells (OPCs) have great potential to grow and differentiate into glioma cells. However, recent studies have indicated that in the nervous system, primitive OPCs exhibit a more pronounced and stronger oncogenic tendency than OPCs [48]. BMDMs can promote the phagocytosis of glioma cells, which in turn induces macrophage polarization from the M1 to the M2 phenotype and results in the development of an immunosuppressive TME [49].

Four TFs, namely, CTCF, POLR2A, SIN3A, and SPI1, were found to target the prognostic genes identified in this study. The TF–mRNA network contained 37 nodes and 91 edges. Hypermethylation of CTCF insulators has been shown to cause overexpression of PDGFRA, an oncogene that contributes to tumor proliferation and invasion in glioma. This finding provides novel insights into epigenetic mechanisms underlying the development of glioma [9]. Moreover, recent studies have shown that SPI1 can enhance the activity of tumor stem cell mesenchyme by regulating the PI3K/Akt signaling pathway, thereby promoting cancer development. This finding suggests that SPI1 is a promising therapeutic target for glioma [50].

In conclusion, using scRNA‐seq and other widely used bioinformatic techniques, we developed and validated a risk model based on five prognostic genes linked to mitochondrial and macrophage polarization in glioma. The biological functions, immune microenvironmental features, clinical relevance, and interaction and regulatory networks of the five genes were investigated, and their expression patterns were validated in patient tissue samples in vitro. Altogether, the findings of this study provide a theoretical basis for investigating the pathophysiological mechanisms through which MRGs and MPRGs contribute to glioma development and offer valuable insights into the prognosis and immunotherapy of glioma. However, the functions and signaling pathways of the prognostic genes identified in this study warrant further investigation.

## Author Contributions

Conceptualization, Pengyu Chen and Heping Wang; methodology, Pengyu Chen; software, Pengyu Chen; validation, Pengyu Chen, Yufei Zhang, Siyao Qu and Shaoyi Li; formal analysis, Pengyu Chen; investigation, Yulian Zhang; resources, Yang Yang; data curation, Chuanpeng Zhang; writing – original draft preparation, Pengyu Chen; writing – review and editing, Pengyu Chen; visualization, Yanbo Yang; supervision, Kun He; project administration, Hanhan Dang; funding acquisition, Yanbing Yu. All authors have read and agreed to the published version of the manuscript.

## Ethics Statement

The clinical tissue samples in this research were approved by the Ethics Committee of China–Japan Friendship Hospital. Each patient was fully and clearly informed and voluntarily signed an informed consent form.

## Conflicts of Interest

The authors declare no conflicts of interest.

## Supporting information


**Figure S1.** Differences in risk scores between the subgroups of clinical features.

**Figure S2.** Expression of prognostic genes in the subgroups of clinical features.

**Figure S3.** Number of genes, total number of mRNA molecules, and percentage of mitochondrial genes before quality control in (A) GSM4119531, (B) GSM4119532, (C) GSM4119533, (D) GSM4119535, (E) GSM4119537, (F) GSM4119538, and (G) GSM4119539.

**Figure S4.** Number of genes, the total number of mRNA molecules, and percentage of mitochondrial genes after quality control in (A) GSM4119531, (B) GSM4119532, (C) GSM4119533, (D) GSM4119535, (E) GSM4119537, (F) GSM4119538, and (G) GSM4119539.

**Figure S5.** Visualization of highly mutated genes in (A) GSM4119531, (B) GSM4119532, (C) GSM4119533, (D) GSM4119535, (E) GSM4119537, (F) GSM4119538, and (G) GSM4119539. The left graph shows the top 2000 highly mutated genes and the right graph labels the TOP 10 genes.

**Figure S6.** (A) Two‐dimensional PCA cell distribution. (B) Trends of prognostic gene expression at various stages with neural progenitor cell differentiation. (C) Trends of prognostic gene expression at various stages with oligodendrocyte cell differentiation.

**Table S1.** Glioma‐related pathways enriched in GSEA results.

**Table S2.** Correlation between prognostic genes and differential immune cells.

**Table S3.** Key miRNAs and lncRNAs acquired based on prognostic genes.

## Data Availability

The data that support the findings of this study are available in the University of California Santa Cruz (UCSC) database at http://genome.ucsc.edu These data were derived from the following resources available in the public domain: the University of California Santa Cruz (UCSC) database, http://genome.ucsc.edu Glioma Genome Atlas (CGGA), http://www.cgga.org.cn the Gene Expression Omnibus (GEO) database, https://www.ncbi.nlm.nih.gov/ MitoCarta3.0 database, https://www.broadinstitute.org/mitocarta.
