# Supplementary figures and images for: Construction of a Prognostic Model for Mitochondria and Macrophage Polarization Correlation in Glioma Based on Single‐Cell and Transcriptome Sequencing
Source: CNS Neurosci Ther. 2024 Nov 3;30(11):e70083. doi: 10.1111/cns.70083 (PMC11532235; doi:10.1111/cns.70083)

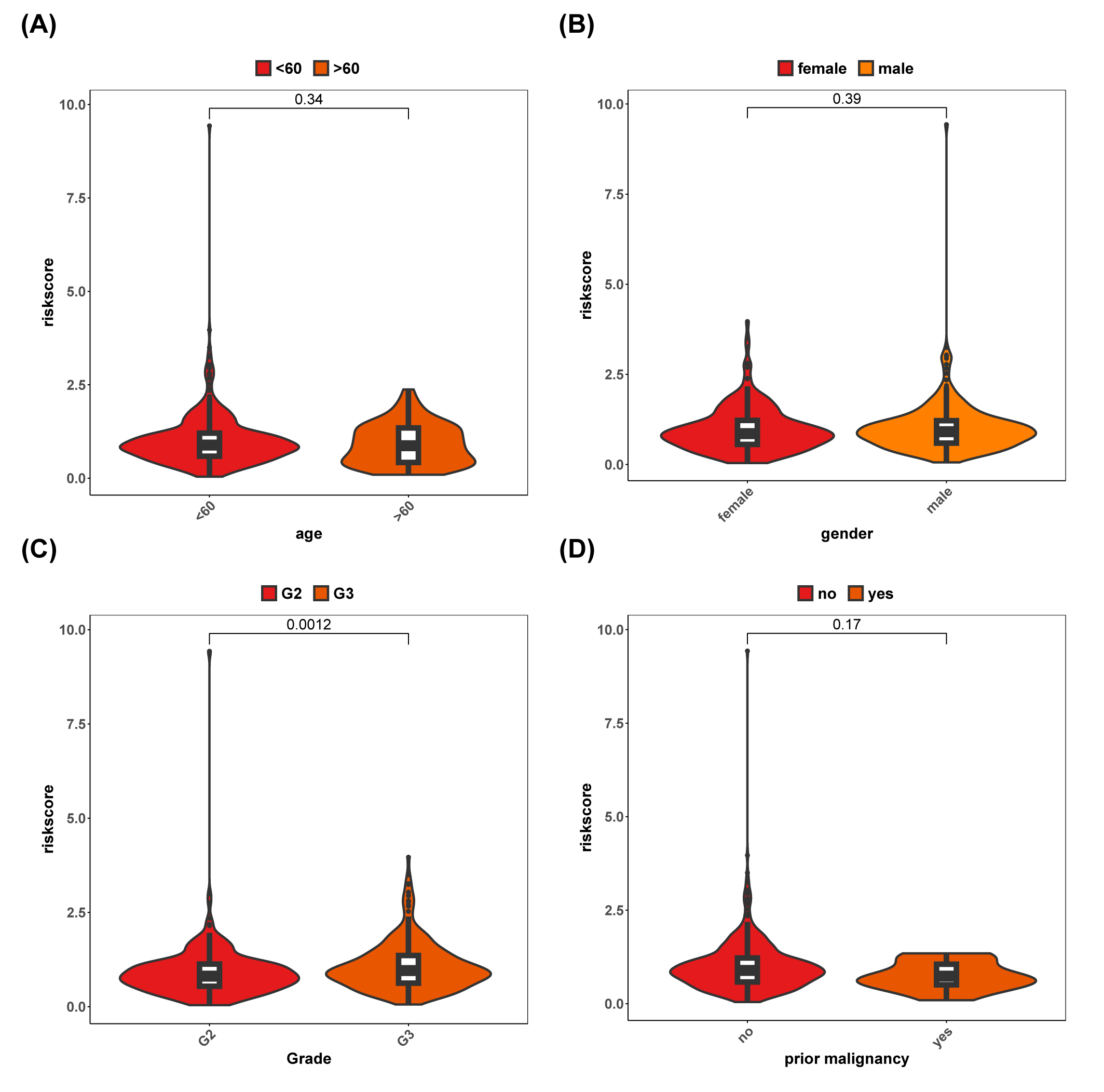

Supplement: Supplementary file 1 — Figure S1. Differences in risk scores between the subgroups of clinical features. [file CNS-30-e70083-s007.tif]

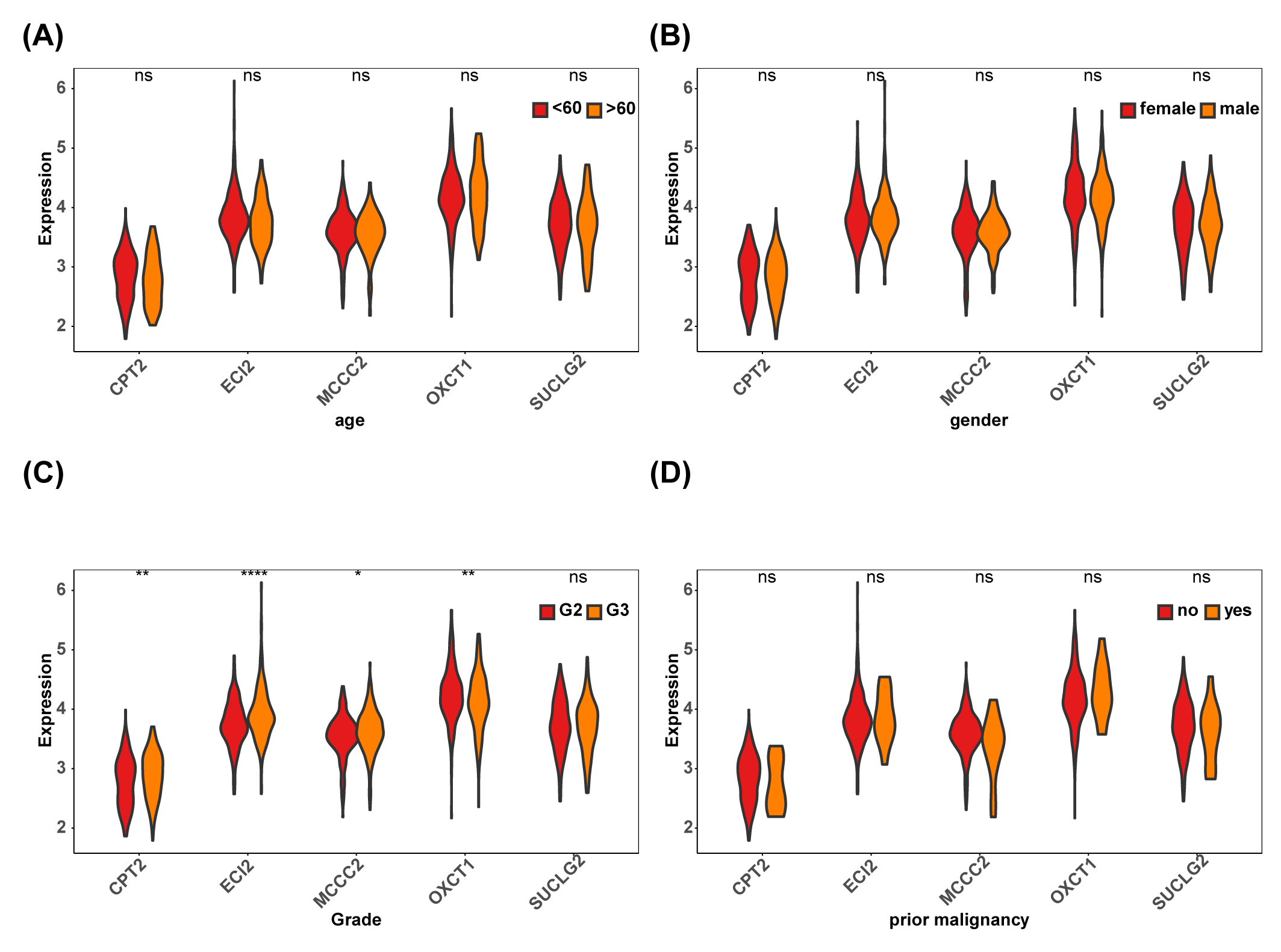

Supplement: Supplementary file 2 — Figure S2. Expression of prognostic genes in the subgroups of clinical features. [file CNS-30-e70083-s006.tif]

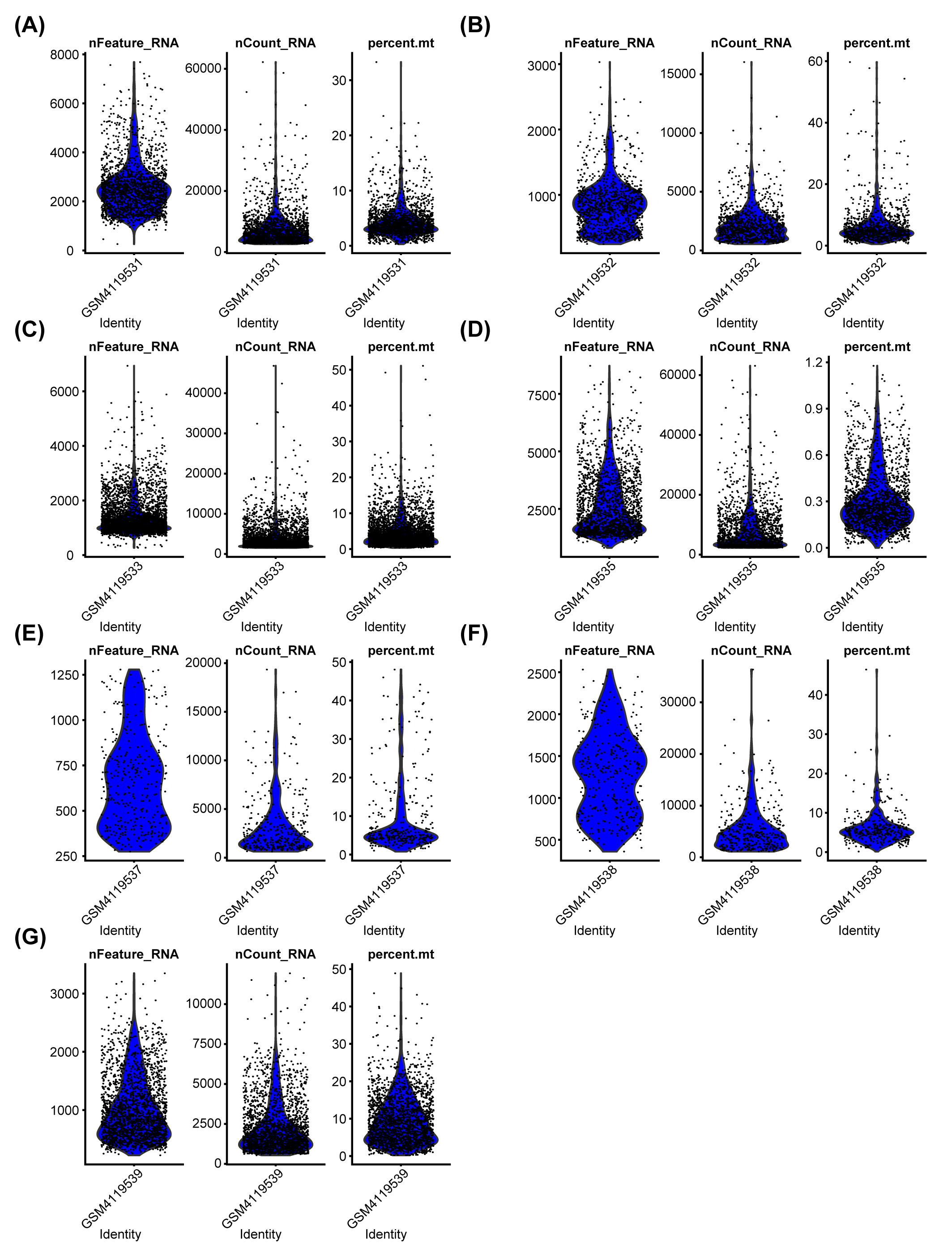

Supplement: Supplementary file 3 — Figure S3. Number of genes, total number of mRNA molecules, and percentage of mitochondrial genes before quality control in (A) GSM4119531, (B) GSM4119532, (C) GSM4119533, (D) GSM4119535, (E) GSM4119537, (F) GSM4119538, and (G) GSM4119539. [file CNS-30-e70083-s002.tif]

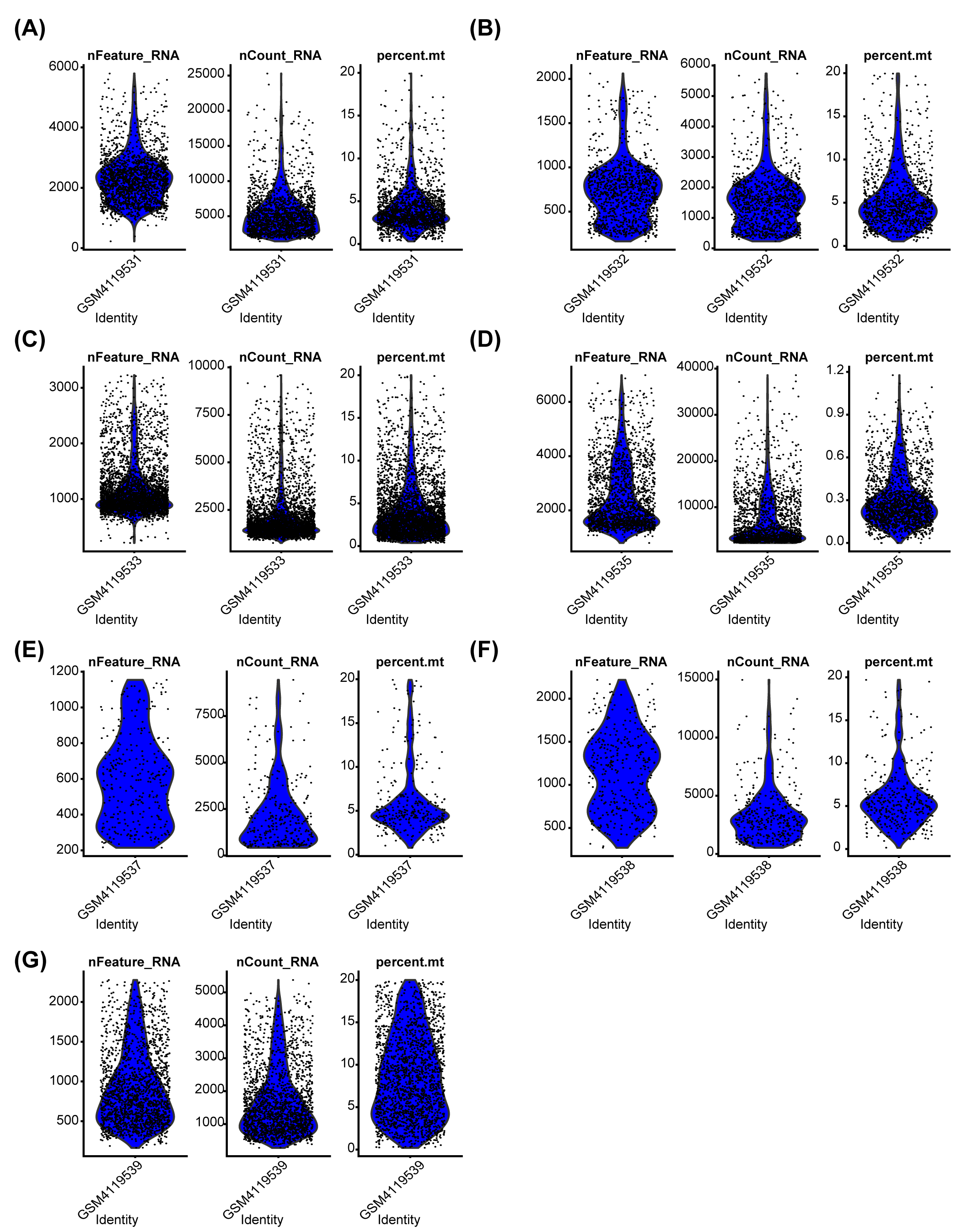

Supplement: Supplementary file 4 — Figure S4. Number of genes, the total number of mRNA molecules, and percentage of mitochondrial genes after quality control in (A) GSM4119531, (B) GSM4119532, (C) GSM4119533, (D) GSM4119535, (E) GSM4119537, (F) GSM4119538, and (G) GSM4119539. [file CNS-30-e70083-s008.tif]

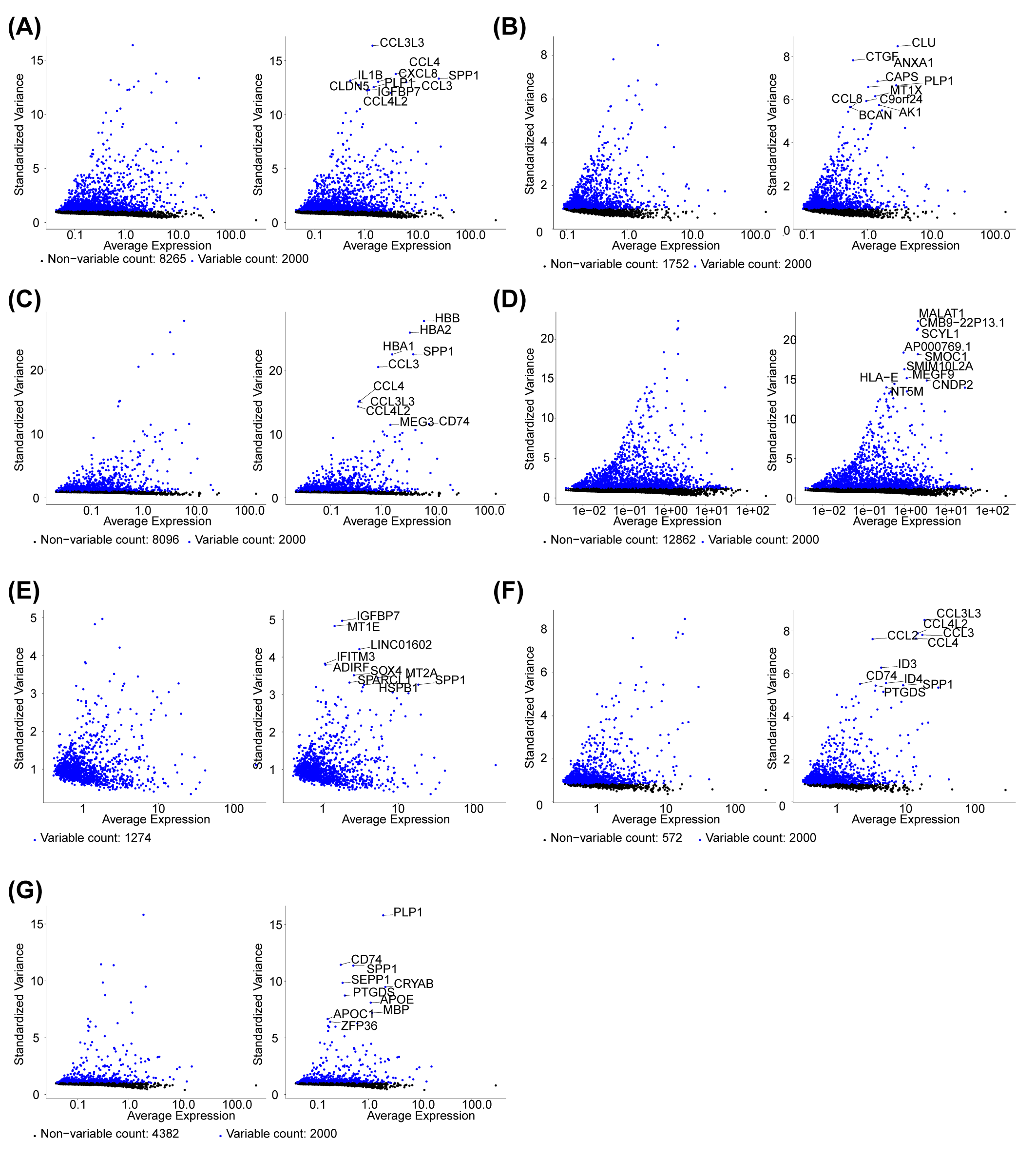

Supplement: Supplementary file 5 — Figure S5. Visualization of highly mutated genes in (A) GSM4119531, (B) GSM4119532, (C) GSM4119533, (D) GSM4119535, (E) GSM4119537, (F) GSM4119538, and (G) GSM4119539. The left graph shows the top 2000 highly mutated genes and the right graph labels the TOP 10 genes. [file CNS-30-e70083-s009.tif]

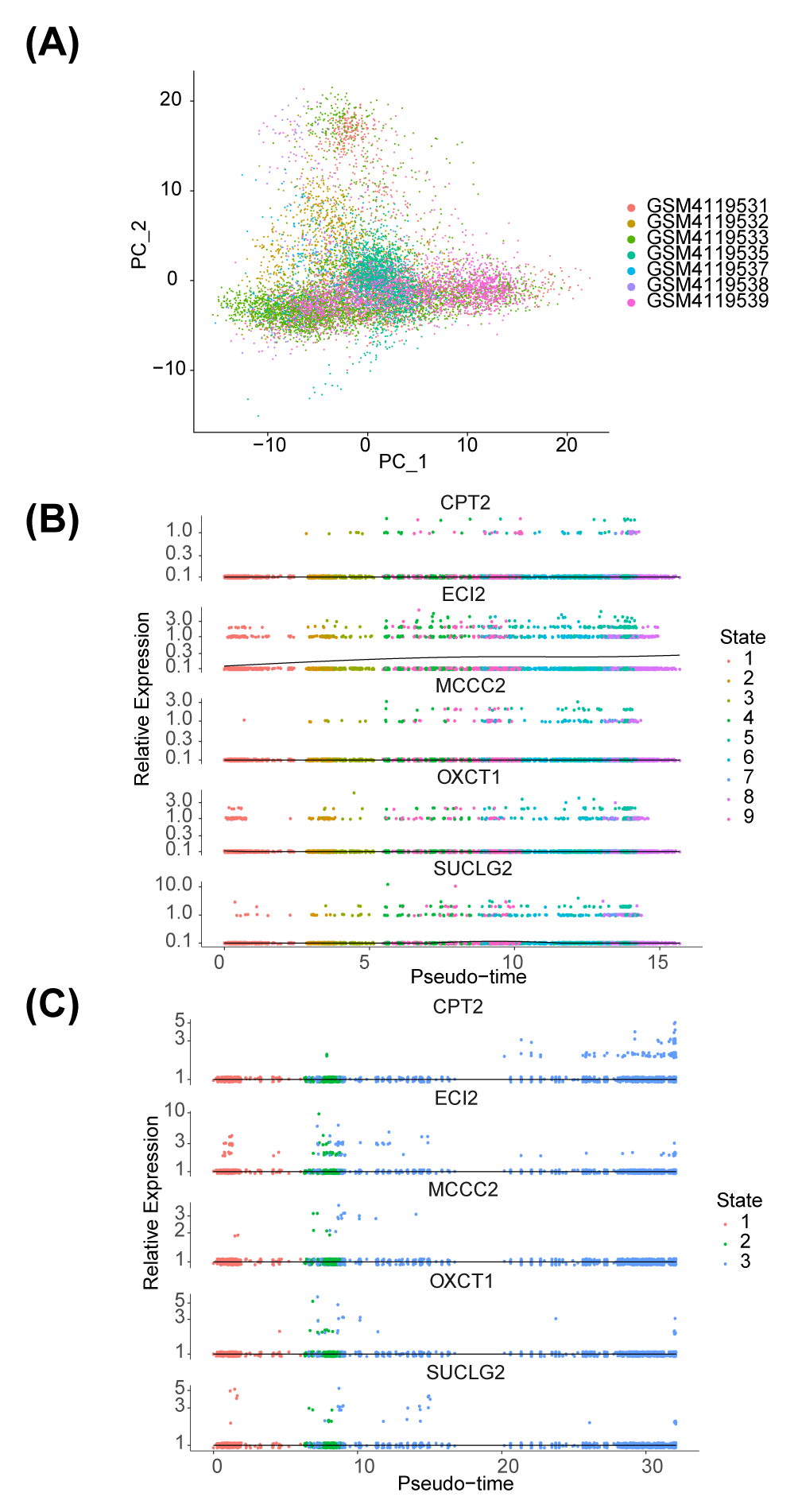

Supplement: Supplementary file 6 — Figure S6. (A) Two‐dimensional PCA cell distribution. (B) Trends of prognostic gene expression at various stages with neural progenitor cell differentiation. (C) Trends of prognostic gene expression at various stages with oligodendrocyte cell differentiation. [file CNS-30-e70083-s005.tif]
