# Supplementary material for: Construction of a Prognostic Model for Mitochondria and Macrophage Polarization Correlation in Glioma Based on Single‐Cell and Transcriptome Sequencing
Source: CNS Neurosci Ther. 2024 Nov 3;30(11):e70083. doi: 10.1111/cns.70083 (PMC11532235; doi:10.1111/cns.70083)
Supplement: Supplementary file 9 — Table S3. Key miRNAs and lncRNAs acquired based on prognostic genes. [file CNS-30-e70083-s001.docx]

| mRNA | miRWalk  （miRNA） | Starbase  （miRNA） | key miRNA | miRNet  （lncRNA） | Starbase  （lncRNA） | key lncRNA |
| --- | --- | --- | --- | --- | --- | --- |
| CPT2 | 212 | 2 | 0 | 0 | 0 | 0 |
| SUCLG2 | 382 | 55 | 9 | 381 | 270 | 66 |
| ECI2 | 54 | 17 | 2 | 48 | 33 | 10 |
| MCCC2 | 402 | 70 | 12 | 564 | 423 | 131 |
| OXCT1 | 437 | 78 | 6 | 294 | 214 | 60 |
